# Pure and Highly Nb-Doped Titanium Dioxide Nanotubular Arrays: Characterization of Local Surface Properties

**DOI:** 10.3390/nano7120456

**Published:** 2017-12-18

**Authors:** Monika Kwoka, Vardan Galstyan, Elisabetta Comini, Jacek Szuber

**Affiliations:** 1 Institute of Electronics, Silesian University of Technology, 44-100 Gliwice, Poland; Jacek.Szuber@polsl.pl; 2 Sensor Lab., Department of Information Engineering, University of Brescia, 25133 Brescia, Italy; vardan.galstyan@ing.unibs.it (V.G.); elisabetta.comini@unibs.it (E.C.)

**Keywords:** TiO_2_ nanotubes, Nb-doping, surface chemistry, XPS, surface morphology, SEM

## Abstract

This paper presents the results of studies of the local surface properties of pure and highly Nb-doped (12 wt %) TiO_2_ nanotubes (TNT) using the X-ray photoelectron spectroscopy (XPS) and scanning electron microscopy (SEM) methods, respectively. XPS analysis showed that the pure TNT exhibit an evident over-stoichiometry combined with high level of undesired C contaminations, which was confirmed by the relative concentration of specific elements O, Ti and C (with respect to all the surface atoms) equal to 0.46, 018 and 0.36, respectively. In turn, for the highly Nb-doped (12 wt %) TNT, a slightly different surface chemistry was observed because the relative concentration of specific elements O and Ti and, with respect to all the surface atoms, is slightly lower, that is, 0.42 and 0.12, respectively; this is directly related to the fact that Nb atoms appeared having the relative concentration at the level of 0.09, whereas the undesired C contaminations reached the same level (0.36), as is the case of pure TNT. In addition, SEM analysis confirms that there are evident free spaces between the specific slops containing several TNT, what was additionally confirmed by the contribution of specific surface bonding coming from the SiO_2_/Si substrate. The obtained information allowed us a new insight on the potential origin of aging effect at the surface of TNT in atmosphere being the undesired limitation for their potential application as the chemical resistive type sensors or in any other fields of their application related to their surface activity.

## 1. Introduction

In the last several years there was an increasing attention of the scientific community to titanium dioxide (TiO_2_), an n-type wide band-gap stable nontoxic oxide semiconductor material, exhibiting extraordinary physical, chemical, electronic, electrochemical and photoactive properties. This was among the reasons that TiO_2_ has been found to be a very important electronic material for potential application in photovoltaics [1,2,3], photocatalysis [4,5], and chemical/gas sensing [6,7,8,9,10].

It is well known that TiO_2_ based gas sensors are particularly attractive for the detection of reducing gases, and that larger surface to volume ratio provides better gas sensing performances [11]. This is why special attention was given to the different forms of nanostructures having large specific surface area. In particular, hierarchical nanostructured TiO_2_ seems to be among the best candidates for the development of gas sensors because its conductance/resistance changes with the surface adsorption and desorption of gas molecules in a reversible way. This is related to the electronic transfer that occurs between the chemical species and the semiconductor upon the molecules adsorption over the surface [12].

Because the conductance change strongly depend on the nanostructures shape and size, nanostructured titania with tubular shape seems to be one of the most promising materials for the fabrication of novel type gas sensing devices [13].

Lately it was also recognized that anatase phase of TiO_2_ is more reactive than the rutile phase [14]. Thus, in order to enhance TiO_2_ sensor properties the phase transformation from anatase to rutile should be avoided, stabilizing the anatase phase even at higher temperatures and hindering its transition to rutile observed already at ~600 °C.

It is also well known that addition of pentavalent atoms like Nb or Ta to titania evidently diminishes the anatase to rutile transformation and also hinders the grain growth [15]. This is extremely important because the addition of different metals as dopants to the metal oxides lattice cause the enhancement of their gas sensing performances (sensitivity, selectivity, response time, etc.) [16].

The most popular methods for the synthesis of one-dimensional TiO_2_ nanostructures are the sol-gel template [17] and the hydrothermal method [18]. Apart from them, to obtain TiO_2_ nanotubular arrays for the development of TiO_2_ nanotubes gas sensors, extremely sensitive towards hydrogen [19] the anodization method was lately develop by our group. Moreover, we have also found that the sensing performances of TiO_2_ nanotubes, in terms of baseline conductivity, response and stability [20,21,22], can be improve by adding of niobium (Nb).

However, a fact that has been well known for over that 20 years should be taken into account, namely, that the transduction of chemical information into the electrical signal change of metal oxides responsible for sensing performances of TiO_2_ nanotubes takes place within the surface space charge layer within the Debye length (about several nm) [23]. 

Thus, it is absolutely crucial to try to propose a new insight into the surface properties of pure and Nb-doped TNT, with a special emphasis on their surface chemistry (nonstoichiometry), including the undesired C surface contaminations, which mainly cause the sensor aging effect after their exposure to atmosphere, something that was until now rather completely ignored in the available literature. This type of information can be obtained by a surface sensitive method, such as X-ray photoelectron spectroscopy (XPS), exhibiting the information depth comparable with the above mentioned Debye length.

According to the above the aim of this research was to obtain the fundamental information on the local surface properties of pure titanium oxide TiO_2_ nanotubes and highly Nb-doped (12 wt %) TiO_2_ nanotubes (TNT), including surface chemistry (stoichiometry/nonstoichiometry, as well as surface contaminations including C species) by using the XPS method, in relation to their surface morphology and additionally verified by Scanning Electron Microscopy (SEM) method.

## 2. Results and Discussion

As mentioned above at the first stage, the XPS studies have been performed in order to check the surface chemistry of prepared pure and highly Nb-doped (12 wt %) TiO_2_ nanotubes (TNT).

**Figure 1:**
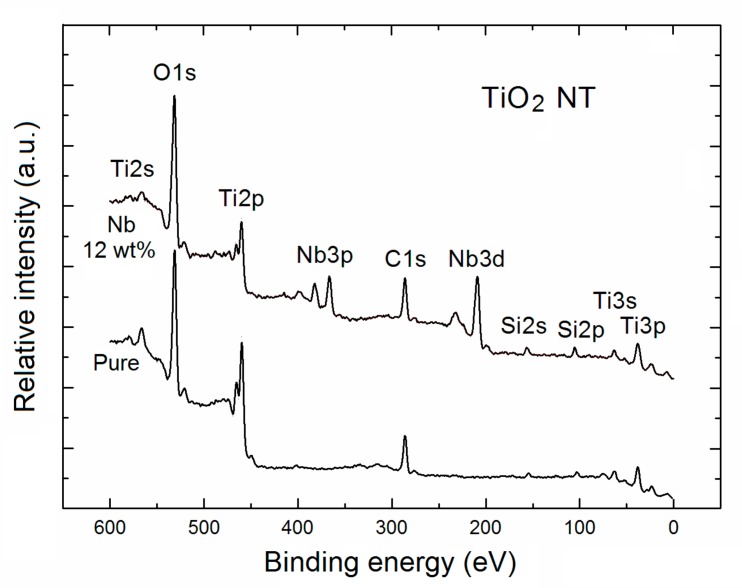
X-ray photoelectron spectroscopy (XPS) survey spectra of pure and Nb-doped TiO_2_ nanotubes.

At the beginning the XPS the survey spectra of pure and highly Nb-doped TNT have been recorded. The respective survey XPS spectra in the limited binding energy (600 eV) are shown in Figure 1.

As can be seen from Figure 1, for the pure TNT the following main core-level XPS peaks of basic elements of TiO_2_, that is, single O1s, as well as, double Ti2p, and single Ti2s, Ti3s and Ti3p peaks are visible. Apart from them there also the core-level XPS peaks of C1s, as well as Si2s and Si2p. It means that from one side, there is an evident strong surface C contamination at the surface of pure TNT, and from the second one, that Si substrate covered by SiO_2_ is not fully covered by TNT, something was completely ignored in the recent paper of Xu et al. [24].

In turn, for the Nb-doped TNT there are additional contributions from the core-level XPS peaks of Nb, that is, single Nb3s, double Nb3p, double Nb3d, single Nb4s, and single Nb4p. Moreover, it was evident that for the Nb-doped TNT the surface C contamination at the similar level as for the case of the pure TNT.

Based on the respective experimental XPS survey spectra (shown in Figure 1) the quantitative analysis of surface chemistry of pure and Nb-doped TNT (within the escape depth of inelastic mean free path of photoelectrons ~3 nm) have been performed. 

It mainly consists in determination of the relative concentration of respective surface atoms like Ti, O, C and Nb, with respect to all the surface atoms using the commonly known formula:

$$n_{i}=\frac{\frac{I_{i}}{ASF_{i}}}{\sum_{i}\frac{I_{i}}{ASF_{i}}}$$

based on the relative intensity (height) *I_i_* of the O1s, Ti2p, Nb3d and C1s core-level lines (peaks) corrected by the transmission function T(E) of electron analyzer CHA PHOIBOS 100 (SPECS, Berlin, Germany) of 1.02, 0.99, 0.88, and 0.90, respectively, as well as by the atomic sensitivity factors (*ASF_i_*) related to the height of specific peaks of O1s (O.66), Ti2p (1.2), Nb3d (1.57), and C1s (0.25), respectively [25,26,27].

Results on the determination of relative concentration of specific elements (with respect to all the surface atoms), and the corresponding partial concentration (in %) of the basic elements of pure and highly doped (12 wt %) Nb-TNT are summarized in Table 1.

For the pure TNT the relative concentration of specific elements O, Ti and C, with respect to all the surface atoms, are equal to 0.46, 018 and 0.36, respectively. What is crucial is that the corresponding partial surface concentration of these elements is 46, 18 and 36%. It means that there is an evident over-stoichiometry of TNT combined with an undesired high partial C concentration.

Slightly different surface chemistry was observed for the highly Nb-doped (12 wt %) TNT. The relative concentration of specific elements O and Ti and with respect to all the surface atoms is slightly lower, 0.42 and 0.12, which is related to the fact that Nb atoms appeared to be having the relative concentration with respect to all the surface atoms at the level of 0.09. It confirms that for this sample even higher nonstoichiometry was observed, whereas the C contaminations reached the same level (0.36), as was the case of pure TNT. 

It means that the concentration of Nb atoms at the surface of Nb-doped TNT is slightly evidently lower with respect to the bulk. It is direct proof that at the surface/subsurface region for both TNT samples some kind of a specific competition is observed between the main matrix atoms like Ti, O and Nb, and undesired C atoms being the main surface contaminations.

This last information is absolutely crucial when trying to apply of Nb-doped TNT as the gas sensors material, which will be more precisely analyzed below. What is also crucial is that the above-described information on the surface chemistry (including stoichiometry) of the pure and Nb-doped TNT derived from XPS survey spectra are in a good correlation with those obtained after precise analysis of the shape of respective XPS main core level O1s, Ti2p, C1s and Nb3d spectral lines using the deconvolution procedure, which is described in detail below.

**Figure 2:**
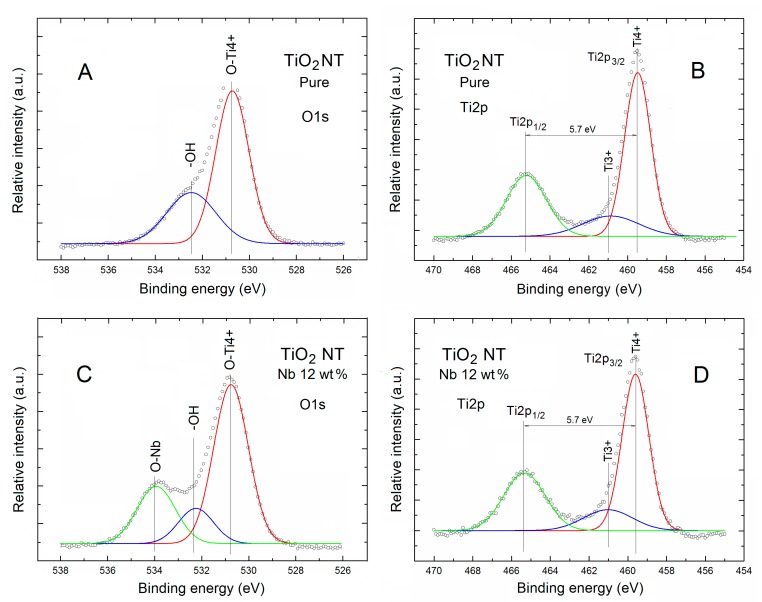
XPS O1s and double Ti2p spectral lines of pure (**A**,**B**) and Nb-doped (**C**,**D**) TNT, respectively, after Gauss deconvolution. The circles correspond to the experimental curves after linear smoothing, whereas the color lines correspond to the respective deconvoluted components.

Figure 2 shows the XPS O1s and double Ti2p spectral lines of pure and Nb-doped TNT after deconvolution using Gauss fitting procedure. 

As has been mentioned earlier, for Nb-doped TNT higher nonstoichiometry of the main matrix TiO_2_ was observed with respect to the pure ones. It means that one can expect different O surface bonding for both samples. This has been confirmed in our detailed analysis of the XPS O1s spectral lines after Gauss deconvolution procedure, as shown in Figure 2 (left column).

For the pure TNT, only two O components were recognized, as shown in Figure 2A. 

The first one (as red), higher in intensity, is located at about 530.5 eV, can be attributed to the lattice oxygen bonded to O-Ti^4+^ bonding at the surface of pure TNT, which was in a good agreement, among others, with the TNT array by Mahajan et al. [28], as well as with the TiO_2_ thin films by Mohanta et al. [29].

The second one (as blue), lower in intensity, is located at about 532.5 eV, can be attributed to the different forms of surface hydroxyl bonding like –OH that can be present at the pure TNT surface after air exposure, which was observed for the highly porous TiO_2_ thin films with nanocrystalline framework by Kondalkar et al. [30]. 

However, this contribution can also reflects the presence of oxygen from carboxyl and alcohol groups adsorbed at the TiO_2_ surface that cannot be also excluded, as suggested by Gao et al. [31]. Moreover, it cannot also be excluded that this component is related partially to the contribution of SiO_2_ covering the Si substrate ,as well hydroxyl groups adsorbed at the SiO_2_ surface (having similar binding energies [32]), because at least TNT were deposited on such SiO_2_/Si substrate.

An evidently different shape of XPS O1s spectral line was observed for the Nb-doped TNT, as shown in Figure 2C. Apart from the two components observed above, in this case an evident component appeared (as green) at binding energy of about 534 eV, which can be attributed to the Nb oxides in form of diametric niobium oxide O–Nb=O, as observed recently by Chennakesavulu et al. [32] at the surface of an Nb_2_O_5_/ZnO catalyst. 

**Figure 3:**
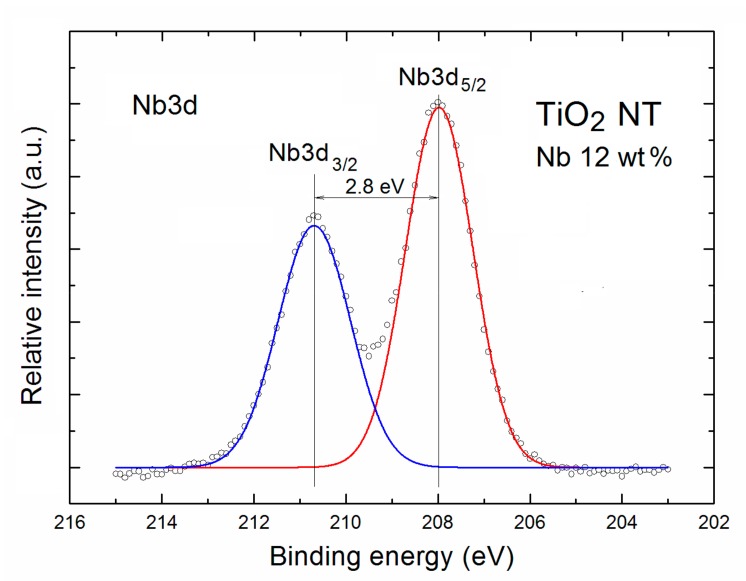
XPS Nb3d spectral line of Nb-doped TNT after Gauss deconvolution. The circles correspond to the experimental curves after linear smoothing, whereas the color lines correspond to the respective spin orbit doublet.

This last information is in a good correlation with the shape of spin orbit doublet XPS Nb3d spectral line of Nb-doped TNT after the Gauss deconvolution procedure shown in Figure 3, which will be discussed in more details below. 

In relation to analysis of components of XPS O1s spectral lines shown in Figure 2A,C at the next step a detailed analysis of the XPS Ti2p spectral line of the pure and Nb-doped TNT was performed.

What is shown in Figure 2B,D for both TNT samples the XPS Ti2p core-level spectral lines contain the spin orbit doublet at binding energy of 459.6 eV (Ti2p_3/2_) (as red) and at a binding energy about 465.3 eV (Ti2p_1/2_) (as green) with the separation binding energy of 5.7 eV and intensity ratio of 0.5, which was in a good agreement with data obtained recently by Xu et al. [24]. 

In order to verify additionally an existence of any potential Ti ions of different valences in both TNT samples the Gauss deconvolution fitting procedure was applied for the higher Ti2p_3/2_ spectral lines, and for both samples two components were observed, as shown in Figure 2B,D. 

A first one located at 459.5 eV can be assigned to the main Ti4+ bonding in TiO_2_, whereas a second one located at about 460.9 eV can be attributed to the Ti3+ bonding, probably related to the unsaturated Ti3+ adjacent to oxygen vacancy, as also observed for the titanium/silica nanostructured system exposed to molecular oxygen by Martinez-Mendez et al. [33], for the Nb-doped TiO_2_ thin films by Tucker et al. [34], and for the self-aligned TNT arrays by Anthony et al. [35]. It should be noted at this moment that the related intensity (area) of respective [Ti3+]/[Ti4+] components was equal to 0.27. 

What is important the binding energy values of the above-described Ti components observed in our studies are in a good agreement with the values reported for various forms of TiO_2_ in the NIST database [36]. It should be additionally noted at this moment that, in our present study of the pure TNT, the existence of Ti2+ state of was not identified. 

The only small difference for the Nb-doped TNT was in the related intensity (area) of the respective components [Ti3+]/[Ti4+] because it slightly decreased to the value of 0.23. This means that it differs only by about 15%, whereas the difference between the partial concentration of Ti in both samples was at the level of 35%; this was also summarized in Table 1. 

As was mentioned above for the Nb-doped TNT, the additional evident component appeared in the XPS O1s spectral line with a binding energy of about 534 eV, which has been attributed to the Nb oxides in the form of diametric niobium oxide O–Nb=O. 

This information was in a good correlation with the shape of respective XPS Nb3d spectral line after the Gauss deconvolution procedure shown in Figure 3.

It contains the spin orbit doublet at a binding energy of 207.9 eV (Nb3d_5/2_) and at a binding energy about 210.7 eV (Nb3d_3/2_), respectively, with the separation binding energy of 2.8 eV, and the intensity ratio of 0.72. 

The obtained binding energies of both Nb components were in a good agreement with the data recently published, among other, Mohanta et al. [29] and by Tucker et al. [34], as well as with data available in the NIST database [36]. The Nb3d_5/2_ components observed in our studies confirm the existence of the pure niobium oxides Nb_2_O_5_ [37] at the surface of Nb-doped TNT.

**Figure 4:**
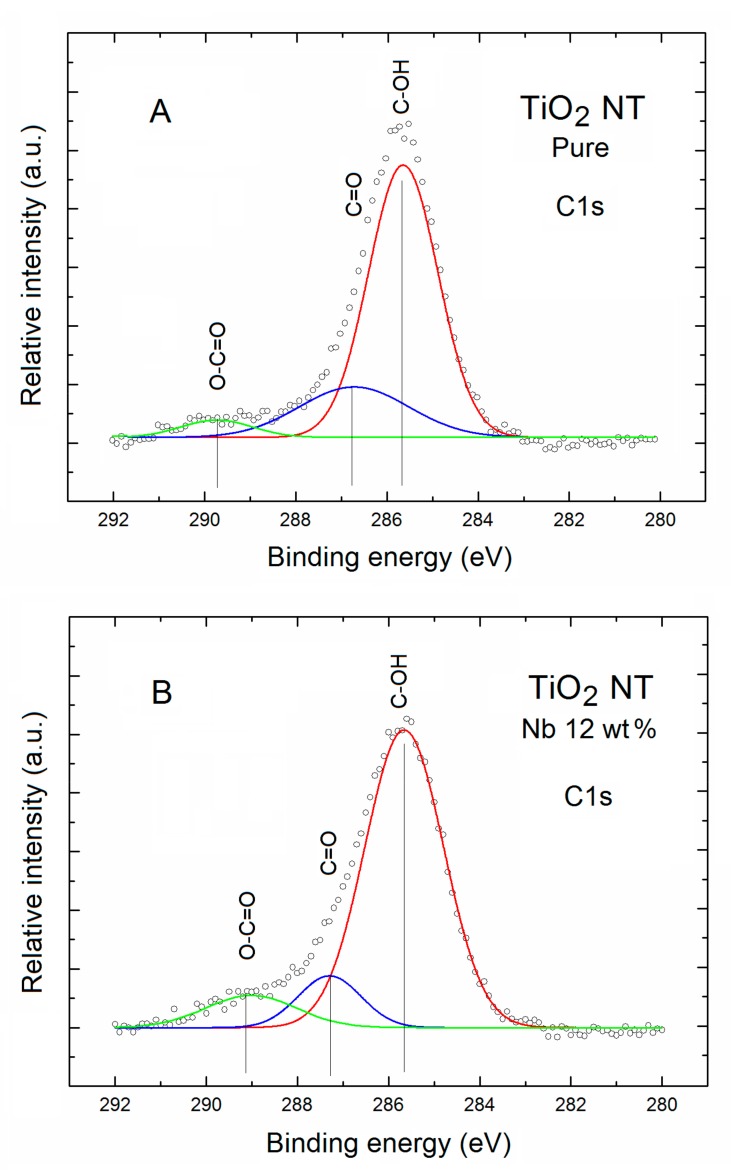
XPS C1s spectral lines of (**A**) pure and (**B**) Nb-doped TNT after the Gauss deconvolution. The circles correspond to the experimental curves after linear smoothing, whereas the color lines correspond to the respective deconvoluted components.

At this point it should be underlined that the potential existence of the hydroxyl groups at the surface of TNT after air exposure is in a good correlation with the fact that their existence was confirmed by the specific component in the XPS C1s spectral lines after the Gauss deconvolution procedure, as shown in Figure 4.

As shown in Figure 4A,B for both TNT samples XPS C1s spectral lines contain three evident components. 

A first one, evidently highest in intensity, is located at binding energy about 286 eV, and can be attributed to the C bonding with water vapor components like C–OH at the surface of TNT after air exposure since this type of bonding are commonly observed at the oxide surfaces. 

In turn, a second one, of evidently lower intensity (only 30% of the first one), located at the binding energy about 287 eV, can be attributed to the C bonding with oxygen surface contamination like C=O also commonly observed at oxide surfaces after air exposure. 

Finally, a third one, of evidently lowest intensity (only 18% of the first one), located at the binding energy about 288 eV, can be attributed to the another C bonding with oxygen surface contamination like O–C=O, also commonly observed at oxide surfaces after air exposure. What is important is that the similar types of C surface bonding were also observed for the anodic oxide formed on Ti–Nb–Sn alloy by Ohtsu et al. [38], at the surface of Nb-doped TiO_2_ thin films prepared by the sol-gel method by Atashbar et al. [39], and at the surface of anatase TiO_2_ nanoparticles by Karthick et al. [40].

As mentioned above, also for the Nb-doped TNT three evident C components were observed with only small shift in binding energy of the second and third components. Moreover, an evident variation of the intensity of second and third components was observed, that is, they exhibit only 14% and 12% of the first one, what means that the contribution of C=O surface bonding was evidently smaller. 

**Figure 5:**
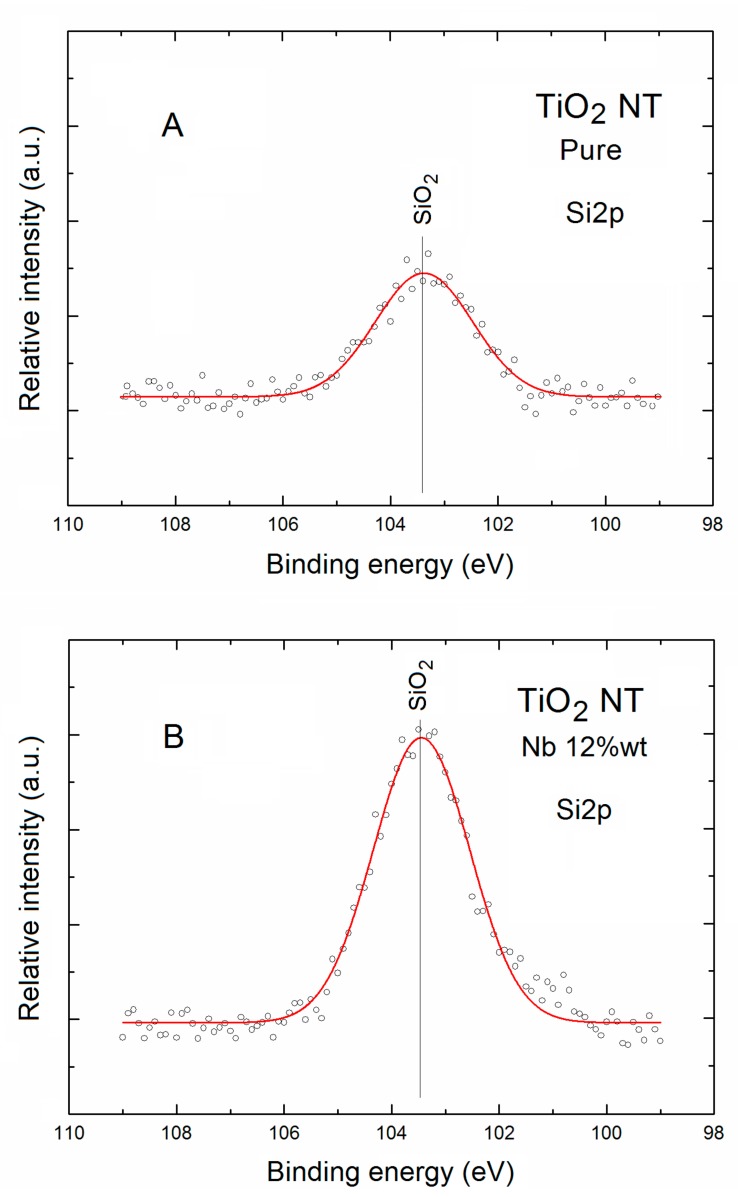
XPS Si2p spectral lines of (**A**) pure and (**B**) Nb-doped TNT after Gauss deconvolution. The circles correspond to the experimental curves after linear smoothing, whereas the color lines correspond to the respective deconvoluted component.

For the additional deeper analysis of C issue, we had in mind to consider the ion sputtering to remove the topmost layer of our TNT samples. However, this procedure looks rather doubtful because of two reasons:

Firstly, during the sputtering the ion Ar^+^ beam is commonly used of diameter of ~mm and focused perpendicularly (or at the specific angle to avoid its implantation) at the sample surface. However, in our studies we used the TiO_2_ NTs, which were aligned almost perpendicularly to the Si substrate (as seen in Figure 5). In such a case, what is absolutely crucial is that the ion beam not penetrates across the TiO_2_ NTs, but along their length. Thus, at such configuration, we are not able to obtain any reliable in depth information concerning the C contaminations across the TiO_2_ NTs of cylindrical surface of diameter ~50 nm, having additionally in mind that the diameter of X-ray beam using in XPS studies is ~3 mm.

Secondly, in the relation to our long-term experience in ion depth profiling studies, we had also in mind that during the ion sputtering the additional undesired effect commonly appears related to the fact that the ion Ar^+^ beam commonly generates the so-called “deep craters”, containing at their side surface the atoms not directly related to the atoms located primarily at the specific depth inside the subsurface of sample under investigation. This type of undesired effect was observed in our recent study of the ion depth profiling of SnO_2_ nanolayers deposited by the Laser-enhanced Chemical Vapor Deposition (L-CVD) method [41].

In addition to all the XPS information above on local surface chemistry of pure and Nb-doped TNT, at this point a contribution of Si substrate covered by SiO_2_ in the survey spectra of both samples confirmed by the existence of an evident XPS Si2p spectral lines visible in Figure 1 will be precisely analyzed below.

For the pure TNT the level of signal-to-noise ratio (S/N) for XPS Si2p spectral line was close to 5, whereas for the Nb-doped TNT it was almost two times larger.

In order to check (verify) any surface Si ions of different valences related to the various SiO_2_ substrate surface bonding, the deconvolution fitting procedure was also applied for the XPS Si2p spectral lines for both TNT samples, what was shown in Figure 5. 

As can be seen in Figure 5A,B the XPS Si2p spectral lines for both TNT samples contain only one evident component.

The binding energy location of this component was about 103.5 eV, which can be assigned to the SiO_2_ surface bonding at the Si substrate. The only difference in the deconvoluted XPS Si2p spectra is related in their evidently various relative intensity, clearly visible in Figure 5A,B.

What is important the binding energy of the main SiO_2_ surface bonding at the Si substrate observed in our studies was in a good agreement with the value reported recently by Alam et al. [42], as well as with data available in the NIST database [36]. It should be additionally noted at this moment that, in our present study, for both TNT samples the existence of another states (valences) of Si surface bonding were not identified.

An appearance of the contribution from SiO_2_ substrate in our XPS spectra of both TNT samples can be interpreted on the base of specific surface morphology of both TNT samples deposited on the SiO_2_/Si substrate.

**Figure 6:**
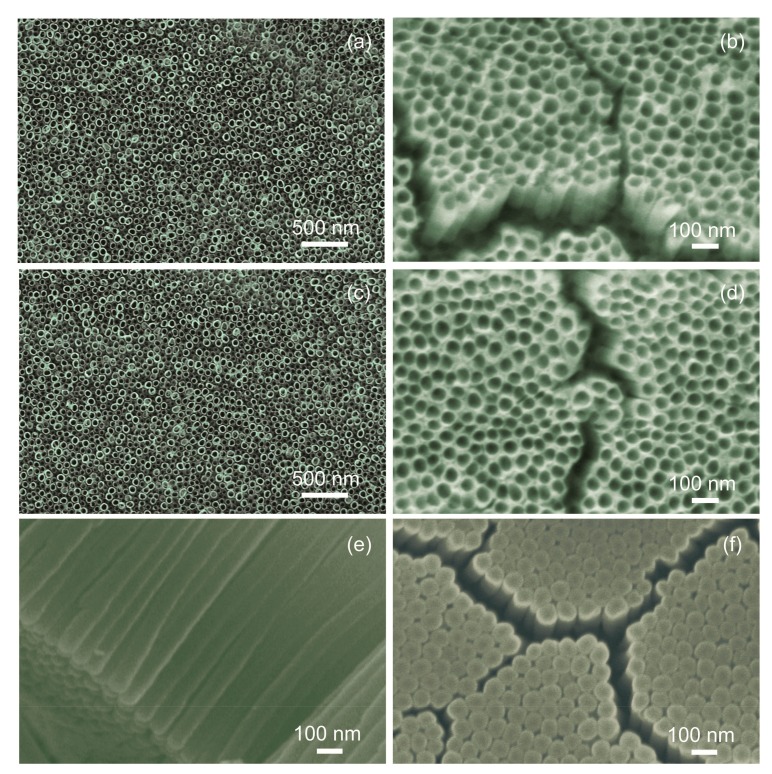
Scanning electron microscopy (SEM) micrographs of the obtained tubular samples: (**a**) and (**b**) the surface morphology of pure TNT arrays with the different magnifications; (**c**,**d**) the surface morphology of 12 wt % Nb-doped TNTs with the different magnifications; (**e**) the cross-sectional view of 12 wt % Nb-doped TNTs, and; (**f**) the bottom-view of 12 wt % Nb-doped TiO_2_ tubular layer.

We have obtained pure and doped TiO_2_ nanotubes with the similar diameters and length by the variation of anodization parameters. Figure 6 shows the SEM micrographs of the obtained pure and 12 wt % doped TNT. The average internal and external diameters of pure and doped nanotubes are 45 and 75 nm, respectively. The length is about 1.7 µm. The relative standard deviation (RSD) is ~15%. The average diameters were determined from the SEM images taken from different locations of the sample and by measuring 50 tubes. The surface morphology (Figure 6a–d), cross-sectional (Figure 6e) and bottom-view (Figure 6f) of the structures show well-aligned and individual nanotubes were grown on Si/SiO_2_ substrates.

However, what is also clearly visible in Figure 6, between the specific slops containing several nanotubes an evident free spaces are observed, which probably originates from the fact that contribution from SiO_2_ covering the Si substrate, as well hydroxyl groups, adsorbed at the SiO_2_ surface (having a similar binding energies [32]) in the respective XPS O1s and XPS Si2p lines was observed, as discussed above.

## 3. Materials and Methods 

Pure (TNT) and highly Nb-doped (12 wt %) TiO_2_ nanotubes (Nb-TNT) have been obtained at the Department of Information Engineering, Brescia University, Italy, using a two-step process.

Within the first step Ti and Ti–Nb films were deposited on Si substrates covered by SiO_2_ by means of RF (13.56 MHz) magnetron sputtering using the targets of Ti and Ti with the holes filled by Nb insets. Within the second step the room-temperature anodization process of Ti and Ti–Nb films was performed using a two-electrode system working at constant voltage mode (potentiostatic mode) using an electrolyte a mixture of 0.3–1 wt % NH_4_F and 0.5–2 mol/L H_2_O in glycerol. We have obtained pure nanotubes in 0.5 wt % NH_4_F and 0.5 mol/L H_2_O containing glycerol. The anodization voltage and time were 120 V and 45 min. Nb-TNT were obtained in 1 wt % NH_4_F and 0.5 mol/L H_2_O containing glycerol. The anodization voltage and time for the preparation of Nb-TNT were 120 V and 35 min.

After anodization the samples were washed in distilled water and dried at room temperature. In order to transfer the deposited amorphous phase to the anatase phase the samples were annealed at 400 °C for 5 h in air. Other experimental details on the preparation of Ti and Ti/Nb films and their anodization one can find in [20,21,22].

The surface chemistry, including contaminations, of the prepared pure and Nb-doped TiO_2_ nanotubes was controlled by XPS method. These experiments were performed at Institute of Electronics, Silesian University of Technology, Gliwice, Poland, using a commercial XPS spectrometer (SPECS, Berlin, Germany) equipped, among others, with the X-ray lamp (XR-50) and a concentric hemispherical analyzer (PHOIBOS-100). The basic working pressure was at the level ~10^−9^ hPa. All the reported binding energies (BE) data have been calibrated to Au4f peak at 84.0 eV. Other experimental details have been described elsewhere [23,41,43].

In turn, the morphological characterization of the above mentioned TiO_2_ NT was carried out at the Department of Information Engineering, Brescia University, Italy, by the SEM method using a LEO 1525 microscope (LEO Electron Microscopy Inc., One Zeiss Drive, Thornwood, NY, USA) equipped with field emission gun.

## 4. Conclusions

The surface chemistry of pure and 12 wt % Nb-doped TNTs deposited on Si substrates covered by SiO_2_ were studied by means of the XPS method. The obtained results were in a good correlation with the samples’ morphological characteristics.

For the pure TNTs the relative concentration of specific elements O, Ti and C, with respect to all the surface atoms, was equal to 0.46, 018 and 0.36, respectively. For the highly Nb-doped (12 % wt) TNT a slightly different surface chemistry was observed since the relative concentrations of specific elements O and Ti, and with respect to all the surface atoms, were slightly lower, 0.42 and 0.12. This is related to the fact that Nb atoms appeared at the surface having the relative concentration with respect to all the surface atoms at the level of 0.09. 

An evident free space was observed between the specific slops containing several TNT by means of SEM analysis, which was additionally confirmed by the contribution from the SiO_2_/Si substrate containing in the respective XPS O1s and XPS Si2p lines as well. 

The obtained results allowed us a new insight on the potential origin of aging effect for TiO_2_ nanotubes in atmosphere being the undesired limitation for their potential application as the chemical sensors. 

## Figures and Tables

**Table 1:** Relative concentration of specific elements (with respect to all the surface atoms), and the corresponding partial concentration (in %) of the basic elements of pure and highly doped (12 wt %) Nb-TiO_2_ nanotubes (TNT).

TNT	Relative Concentration of Specific Elements with Respect to All the Surface Atoms				Partial Surface Concentration of Specific Elements (%)			
	O	Ti	Nb	C	O	Ti	Nb	C
**Pure**	0.46	0.18	0	0.36	46	18	0	36
**Nb-doped**	0.42	0.12	0.09	0.36	42	12	9	36
